# 3D Visualization of Developmental Toxicity of 2,4,6-Trinitrotoluene in Zebrafish Embryogenesis Using Light-Sheet Microscopy

**DOI:** 10.3390/ijms17111925

**Published:** 2016-11-17

**Authors:** Juneyong Eum, Jina Kwak, Hee Joung Kim, Seoyoung Ki, Kooyeon Lee, Ahmed A. Raslan, Ok Kyu Park, Md Ashraf Uddin Chowdhury, Song Her, Yun Kee, Seung-Hae Kwon, Byung Joon Hwang

**Affiliations:** 1 Division of Biomedical Convergence, College of Biomedical Science, Kangwon National University, Chuncheon 24341, Korea; eum1230@naver.com (J.E.); wlswns02@naver.com (J.K.); tjdud729@naver.com (S.K.); lky@kangwon.ac.kr (K.L.); ahmed.raslan8080@gmail.com (A.A.R.); ashraf.rx@gmail.com (M.A.U.C.); yunkee@kangwon.ac.kr (Y.K.); 2 Division of Environmental Research Center, Kangwon National University, Chuncheon 24341, Korea; polarbear723@naver.com; 3 Korea Basic Science Institute, Chuncheon Center, Chuncheon 24341, Korea; okpark81@kbsi.re.kr (O.K.P.); swher@kbsi.re.kr (S.H.); 4 Department of Molecular Bioscience, College of Biomedical Science, Kangwon National University, Chuncheon 24341, Korea

**Keywords:** zebrafish embryos, pink water, trinitrotoluene, single plane illumination microscopy, light-sheet microscopy, 3D live imaging, cardiac defect, fetal defect

## Abstract

Environmental contamination by trinitrotoluene is of global concern due to its widespread use in military ordnance and commercial explosives. Despite known long-term persistence in groundwater and soil, the toxicological profile of trinitrotoluene and other explosive wastes have not been systematically measured using in vivo biological assays. Zebrafish embryos are ideal model vertebrates for high-throughput toxicity screening and live in vivo imaging due to their small size and transparency during embryogenesis. Here, we used Single Plane Illumination Microscopy (SPIM)/light sheet microscopy to assess the developmental toxicity of explosive-contaminated water in zebrafish embryos and report 2,4,6-trinitrotoluene-associated developmental abnormalities, including defects in heart formation and circulation, in 3D. Levels of apoptotic cell death were higher in the actively developing tissues of trinitrotoluene-treated embryos than controls. Live 3D imaging of heart tube development at cellular resolution by light-sheet microscopy revealed trinitrotoluene-associated cardiac toxicity, including hypoplastic heart chamber formation and cardiac looping defects, while the real time PCR (polymerase chain reaction) quantitatively measured the molecular changes in the heart and blood development supporting the developmental defects at the molecular level. Identification of cellular toxicity in zebrafish using the state-of-the-art 3D imaging system could form the basis of a sensitive biosensor for environmental contaminants and be further valued by combining it with molecular analysis.

## 1. Introduction

Contamination of natural environments by explosives and their degradation products during field use or manufacturing waste streams exposes diverse organisms, including humans, to potential toxicity. Trinitrotoluene (TNT), a polynitro-organic compound, is one of the main components of explosives and had been detected before in the environment at a concentration of 0.4–21,960 µg/L in ground water, 1.0–3375 µg/L in surface water, 6.7–711,000 mg/kg in sediment, and 0.08–87,000 mg/kg in soil at US military installations, processing facilities and military firing ranges [1].

Acute and chronic exposure of TNT to workers has caused toxicity in the liver, the hematopoietic system and the eye [2,3,4]. Hematoxicity has been examined in rats exposed to TNT, and resulted in hemolysis, destruction of hemoglobin in erythrocytes, decrease in hematocrit value and formation of methemoglobin [5,6,7,8]. Robidoux and colleagues measured the concentration of TNT in soil mesocosms to be from 25 to 17,063 mg/kg [9], and another report showed that the concentration of TNT and its degradation products, 2-amino-4,6-dinitrotoluene (2-ADNT) and 4-amino-2,6-dinitrotoluene (4-ADNT), ranged from sub-microgram to several hundred grams per kilogram within the water system of a pond in an industrial area in Sweden [10]. TNT in soil has been shown to cause lethal and sublethal toxicity in soil invertebrates and freshwater fish and to decrease reproduction rates at concentrations of several hundred mg TNT/kg dry soil [9,11,12,13], as well as in various aquatic species and mammals [1]. Most TNT toxicity tests using animal models have investigated high concentrations of TNT with outcome measures of survival, reproductive rate or behavioral change. It is important that screening tools sensitive enough to detect in vivo biological toxicity of TNT and related compounds at low concentrations are developed.

Pink water is explosive-contaminated wastewater, generated during munition filling or demilitarization operations. The main contaminants of pink water, TNT (trinitrotoluene), RDX (cyclotrimethylenetrinitramine) and HMX (cyclotetramethylene-tetranitramine), are highly resistant to ambient biodegradation and persist in soils and groundwater leading to environmental concerns [11,14]. Despite broad use globally, the toxicity of TNT-contaminated groundwater to living organisms has not been systematically examined in vivo.

Zebrafish (*Danio rerio*) embryos are ideal model vertebrates for assessing the acute toxicity of environmental samples and newly developing compounds [15,16]. Their development has been extensively characterized and embryos remain transparent until late pharyngula stages, allowing unobstructed imaging of key morphological events. Recent advances in imaging technologies have provided opportunities for more sensitive and informative toxicological screening using zebrafish embryos. Single Plane Illumination Microscopy (SPIM)/light sheet microscopy has considerable advantages over conventional microscopy methods, particularly in the context of embryonic development [17,18,19,20,21,22,23,24,25,26]. In SPIM, a millimeter-thick laser sheet is used to illuminate the focal plane; this minimizes sample exposure time, allowing faster throughput and longer-term imaging protocols without compromising image quality. The extended field of view provided by SPIM can help establish interactive relationships between cells and tissues likely to be important during development.

In this study, we visualized the effects of pink water and its main chemical compound TNT on cardiac development and blood circulation in zebrafish embryogenesis at cellular resolution using 3D SPIM. We report dose-dependent toxic effects of TNT on zebrafish pigmentation, and on the developing cardiac and circulatory systems in 3D. These results may be relevant to humans and other vertebrates and demonstrate the advantages of SPIM and the utility of zebrafish embryogenesis as in vivo toxicological assay.

## 2. Results

### 2.1. Pink Water Inhibits Melanin Synthesis and Affects Melanocyte Development

**Figure 1:**
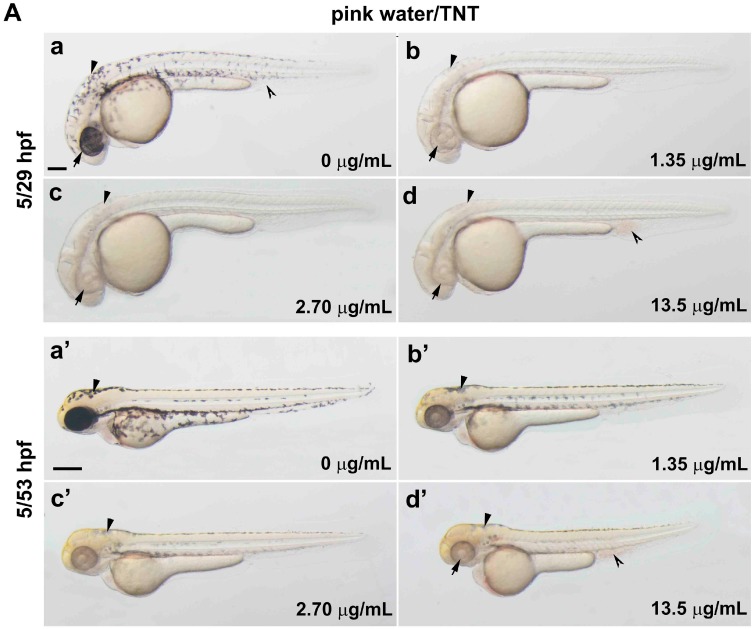

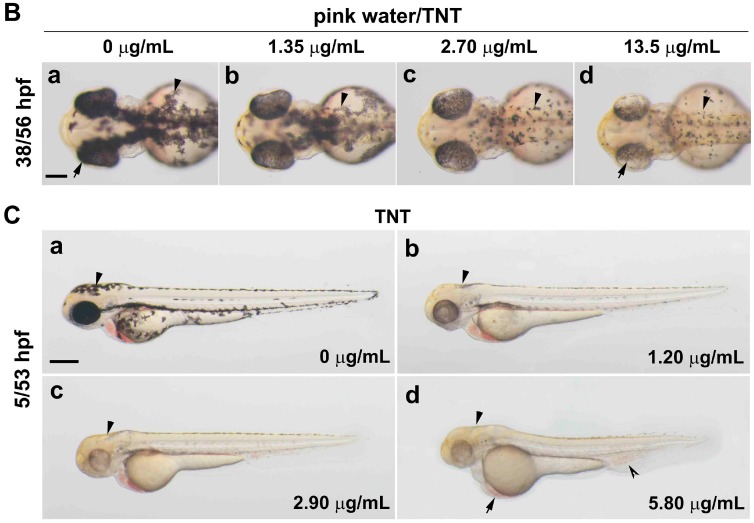
Trinitrotoluene (TNT) in pink water causes developmental defects in zebrafish. (**A**) Bright-field lateral view of embryos treated with pink water. Embryos were exposed to pink water containing TNT at 0 µg/mL (**a**,**a′**), 1.35 µg/mL (**b**,**b′**), 2.70 µg/mL (**c**,**c′**), and 13.5 µg/mL (**d**,**d′**) from 5 till 29 hpf (**a**–**d**, scale bar 250 µm) or 53 hpf (**a′**–**d′**, scale bar 500 µm) and imaged using a stereoscope. Defects in pigmentation were evident at 29 hpf (**a**–**d**, arrow for eye color, arrowhead for body pigment, 3.2× magnification) and at 53 hpf (**a′**–**d′**, arrow for abnormal heart, arrowhead for body pigment, 2.5× magnification). Morphological changes in body shape and a defect in blood circulation (split arrowhead) were observed in embryos treated with pink water containing 13.5 µg/mL of TNT at 53 hpf (**d′**). 5/29 hpf, treatment starting at 5 hpf, observation at 29 hpf; 5/53 hpf, treatment starting at 5 hpf, observation at 53 hpf; (**B**) Dorsal views of embryos treated with pink water from 38–56 hpf. (**a**–**d**) Embryos show a dose-dependent pigmentation defect. Less pigment is visible in the eye (arrow) and trunk melanocytes (arrowhead). 8× magnification, scale bar 250 µm. 38/56 hpf, treatment starting at 38 hpf, observation at 56 hpf; (**C**) Embryos treated with TNT at 0 µg/mL (**a**), 1.20 µg/mL (**b**), 2.90 µg/mL (**c**), and 5.80 µg/mL (**d**) from 5 hpf had similar phenotypes to embryos treated with pink water, including pigmentation defect (arrowhead), abnormal heart (arrow), and defective blood circulation (split arrowhead). 5/53 hpf, treatment starting at 5 hpf, observation at 53 hpf, scale bar 500 µm.

The explosive content of the pink water used in this study was determined by high-performance liquid chromatography (HPLC) analysis using a YL-9100 HPLC instrument (Young Lin Instrument Co., Anyang, Korea) with TC-C18 column at 30 °C as shown in Materials and Methods (Figure S1). The major peak in the HPLC profile of pink water was verified as TNT (2,4,6-trinitrotoluene) with a concentration 45.54 ± 0.49 µg/mL; RDX (cyclotrimethylenetrinitramine) and HMX (cyclotetramethylene-tetranitramine) were not detected in our samples. To assess toxicity, zebrafish embryos were exposed to dilutions of pink water containing 0, 1.35, 2.70 or 13.5 µg/mL TNT from 5 h post fertilization (hpf), when embryos are at 50% epiboly stage, until 29 (24 h incubation, Figure 1(Aa–d)) or 53 hpf (48 h incubation, Figure 1(Aa′–d′)). When embryos were treated with pink water containing 1.35 µg/mL TNT, pigmentation was almost completely absent at 24 h post-treatment and reduced at 48 h post-treatment compared with controls. Higher concentrations of pink water/TNT resulted in more complete inhibition of pigmentation. These results suggest that pink water may suppress melanin synthesis or melanocyte development during early zebrafish embryogenesis and that the severity of the defect is dose-dependent.

Next, embryos were exposed to pink water from 38 hpf to assess toxicity after the key developmental processes of cleavage, gastrulation and neurulation were complete. Dose-dependent pigmentation defects were observed at 18 h post-treatment: eye pigment (arrow) and trunk melanocytes (arrowhead) were visibly reduced (Figure 1B), although their severity was reduced compared to exposure at 5 hpf. Two possibilities could explain the pigmentation defect caused by pink water exposure: inhibition of melanocyte development or disruption of melanin synthesis. To investigate these possibilities, we carefully examined melanocytes in the embryos at high magnification (Figure 1B). The number of melanocytes and the amount of melanin in each melanocyte was reduced by pink water treatment in a dose-dependent manner. We also noted that following pink water treatment melanin became aggregated in small clusters in the center of pigment cells in a dose-responsive manner. This contrasted with control embryo melanocytes, in which melanin appeared to branch diffusely throughout the cytoplasm. Overall, these data implicate that TNT in pink water may affect early melanocyte development and disrupts normal melanin synthesis and cellular distribution.

### 2.2. TNT in Pink Water Causes Early Developmental Defects in Zebrafish Embryogenesis

Embryos treated with pink water containing 13.5 µg/mL TNT had shorter body length and abnormal development of blood islands compared with controls (Figure 1Ad′). We also observed disrupted cardiac morphogenesis and blood accumulation in the heart and blood islands (Figure 1A), suggesting that blood circulation is disrupted in embryos treated with pink water.

To confirm that the observed toxic effects of pink water were caused specifically by TNT, we treated embryos with known dilutions of TNT in E3 media with final 1% ethanol and tested for biological toxicity. Dose-dependent pigmentation defects were observed in embryos treated with TNT from 5 hpf for 48 h (Figure 1C). Embryos treated with TNT had similar phenotypes to embryos treated with pink water: shorter body length, abnormal development of heart and blood islands, blood accumulation and cardiac defects (Figure 1C). These data indicate that the developmental defects caused by pink water in our experiments are likely to be due to TNT toxicity.

### 2.3. Pink Water Affects Heart Development and Function

**Figure 2:**
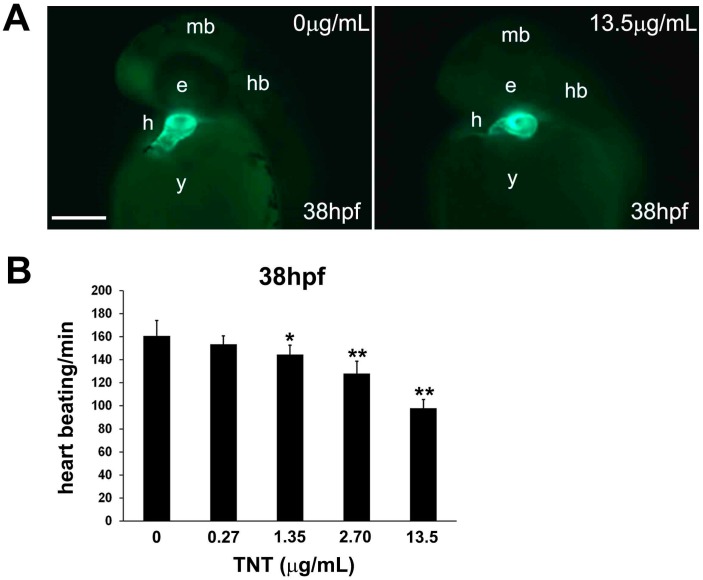
Trinitrotoluene (TNT) in pink water causes defects in heart development and function. (**A**) Tg(cmlc2:EGFP) embryos treated with pink water have myocardiac defects. Embryonic myocardiocytes were imaged at 38 hpf (hours post fertilization) with heads orientated upwards, scale bar 500 µm. Myocardiocytes in the atrium and ventricle were visualized by enhanced green fluorescent protein (EGFP) fluorescence in controls (**a**) and in embryos treated with pink water containing 13.5 µg/mL TNT for 33 h (**b**); (**B**) Heart rate is dose-dependently decreased in 38 hpf embryos by exposure to pink water containing TNT at 0, 0.27, 1.35, 2.70 and 13.5 µg/mL (*n* = 20 each). Following exposure to pink water from 5 hpf, Tg(cmlc2:EGFP) embryonic hearts were imaged laterally at 38 hpf using an Axioimager II fluorescence microscope (Carl Zeiss, Overkochen, Germany). * *p* < 0.05, ** *p* < 0.001.

To understand how pink water affects heart development and function, we investigated its effects on cardiac morphology in more detail. The heart is comprised of the myocardium and endocardium, which originate from different embryonic tissues. We first used the zebrafish transgenic line Tg(cmlc2:EGFP) in which enhanced green fluorescent protein (EGFP) is specifically expressed in cardiac myocytes of the myocardium, allowing live fluorescent imaging of myocardial development [27]. Tg(cmlc2:EGFP) embryos were exposed to pink water from 5 hpf for 33 h and observed using a SZX16 fluorescent stereoscope (Olympus, Tokyo, Japan). At 38 hpf, the myocardium of the atrium and ventricles of embryos treated with pink water containing 13.5 µg/mL TNT were abnormal compared with control embryos (Figure 2A). Time-lapse imaging and measurements of heart rate confirmed that pink water exposure decreased zebrafish heart rate in a dose-dependent manner in the embryos (Figure 2B). These data indicate that pink water may directly affect zebrafish heart physiology in addition to causing morphological defects during development.

**Figure 3:**
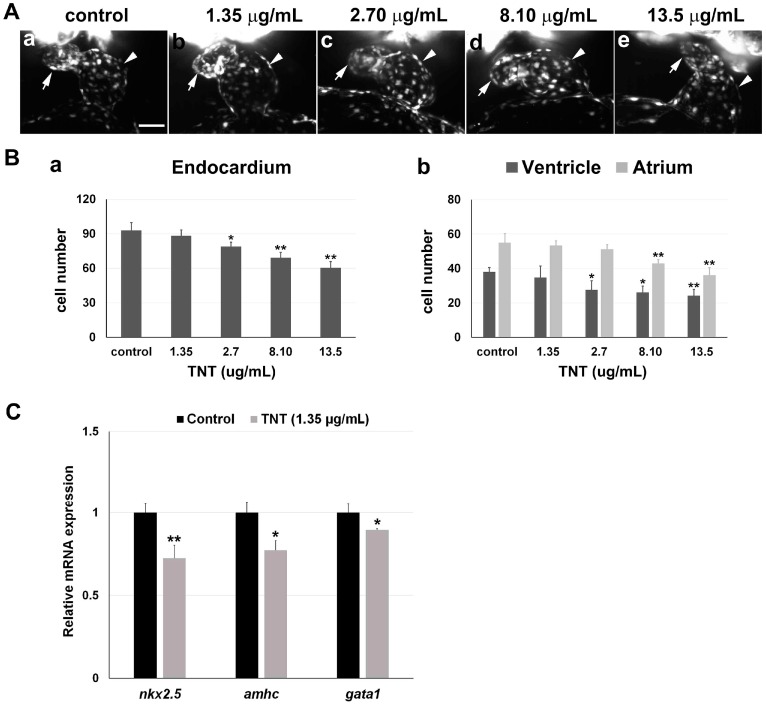
3D light-sheet imaging/SPIM (Single Plane Illumination Microscopy) of TNT cardiac toxicity. (**A**) TNT in pink water caused abnormal cardiac looping in a dose-dependent manner. (**a**–**e**) Endocardiums were visualized by SPIM in 38 hpf Tg(fli1a:EGFP) embryos treated with pink water containing TNT at 0 (**a**, control), 1.35 (**b**), 2.70 (**c**), 8.10 (**d**) and 13.5 µg/mL (**e**) from 5 hpf (2 experiments, *n* = 5 for each treatment). Live 3D reconstructions of the hearts were generated using Arivis software. Arrow, atrium; arrowhead, ventricle. 10× water lens, scale bar 50 µm. 3D reconstruction of each heart imaging is individually shown in Supplementary video 1A–E, respectfully; (**B**) (**a**) Treatment with pink water significantly reduced endocardial cell number in a dose-dependent manner; (**b**) The endocardium in the atrium was affected by TNT toxicity to a greater extent than the endocardium in the ventricle. * *p* < 0.05, ** *p* < 0.001; (**C**) Quantitative real time PCR (qPCR) analysis to measure mRNA expression of two heart-specific genes, *nkx2.5* and *amhc*, and a blood specific gene, *gata1*, in the embryos treated with 0 and 1.35 µg/mL TNT from 5 till 36 hpf. Data are the mean ± SEM of three independent samples, differences between the means were evaluated with an independent-samples *t*-test. * *p* = 0.001, ** *p* < 0.0005.

We next investigated TNT toxicity in Tg(fli1a:EGFP) embryos that robustly express EGFP in the developing endocardium of the heart. Endocardiocytes were visualized by SPIM/light-sheet microscopy in 38 hpf Tg(fli1a:EGFP) embryos treated with pink water containing TNT at 0, 1.35, 2.70, 8.10, and 13.5 µg/mL from 5 hpf (2 experiments, *n* = 5 for each treatment). The result revealed dose-dependent endocardial defects and disrupted cardiac looping of the atrium and ventricle at the cellular level (Figure 3A, Supplementary video 1A–E). Live 3D reconstructions of the hearts were generated using Arivis software 2.10.4. 3D reconstructions of the embryonic endocardium were generated and presented in video format for pink water treatment with TNT at 0 (Supplementary video 2A), 1.35 (Supplementary video 2B), 2.70 (Supplementary video 2C), 8.10 (Supplementary video 2D) and 13.5 µg/mL (Supplementary video 2E). Pink water treatment dose-dependently reduced the total number of endocardial cells in the heart, and it was noted that the atrium was affected to a greater extent than the ventricles (Figure 3B). Our data demonstrate that light-sheet microscopy can reveal pollutant-associated cardiac toxicity at cellular resolution in 3D allowing quantitation of hypoplastic heart chamber formation.

To investigate TNT toxicity at the molecular level, we performed quantitative real-time PCR (qPCR) using primers for two heart-specific genes, *nkx2.5* and *amhc*, and the blood-specific gene, *gata1*, on embryos treated with the lowest TNT dose, 1.35 µg/mL. We found mRNA expression levels of both *nkx2.5* and *amhc* were significantly decreased in the treated embryos at 36 hpf, compared to controls (Figure 3C). *gata1* mRNA expression was also decreased in the treated embryos (Figure 3C). Our results demonstrate that 3D SPIM imaging and qPCR are sensitive enough to detect the toxicity of low concentration of TNT (1.35 µg/mL) on heart development at the cellular and molecular level.

**Figure 4:**
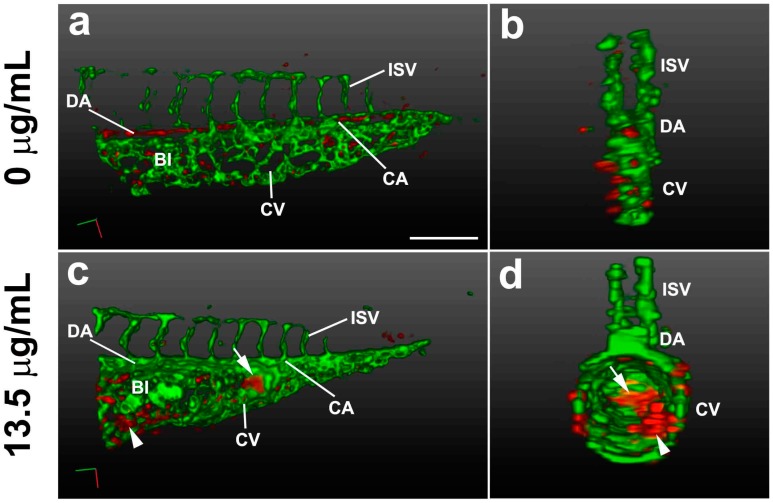
3D light-sheet/SPIM imaging of the TNT circulation defect. The vasculature (green) and blood cells (red) of the posterior trunk were visualized in Tg(gata1:DsRed/fli1a:EGFP) embryos at 38 hpf. 3D reconstructions are shown at lateral view (**a**,**c**) and transverse view (**b**,**d**), using Arivis software (surface function). (**a**,**b**) Embryos treated for 33 h with control E3 media; (**c**,**d**) Embryos treated for 33 h with pink water containing 13.5 µg/mL TNT showing swollen body structure with abnormal blood islands (arrowhead) and blood accumulation (arrow). 20X water lens, 0.8 zoom. Scale bar, 150 µm. BI, blood Island; CA, caudal artery; CV, caudal vein; DA, dorsal aorta; ISV, intersegmental vessel. The 3D movies of the reconstructions are presented in Supplementary video 2A,B.

Live 3D SPIM imaging of Tg(gata1:DsRd/fli1a:EGFP) embryos, which express DsRed (red fluorescent protein) in blood cells under control of the gata1 promoter and EGFP in the developing endocardium and blood vessels under control of the fli1 promoter, revealed wide expansion of the blood islands and caudal vein in the caudal trunk region accompanied by alterations in cell shape when they were treated with pink water containing 13.5 µg/mL TNT (*n* = 20 embryos, 100%). These data suggest that TNT in pink water may disrupt cell–cell interactions between the right and left endothelial walls of the blood island at high concentrations, causing morphological defects (Figure 4, Supplementary video 2A,B). We also noted that blood cells accumulated in the tail by gravity when embryos were orientated vertically (Figure 4c,d, arrow) and were not detected in the dorsal aorta and caudal artery. Clumping of blood cells in blood islands was also observed (Figure 4c,d, arrowhead), indicating defective circulation. This may be the result of reduced blood pumping capacity of the heart following TNT exposure.

### 2.4. Live Imaging of TNT-Associated Cell Death

**Figure 5:**
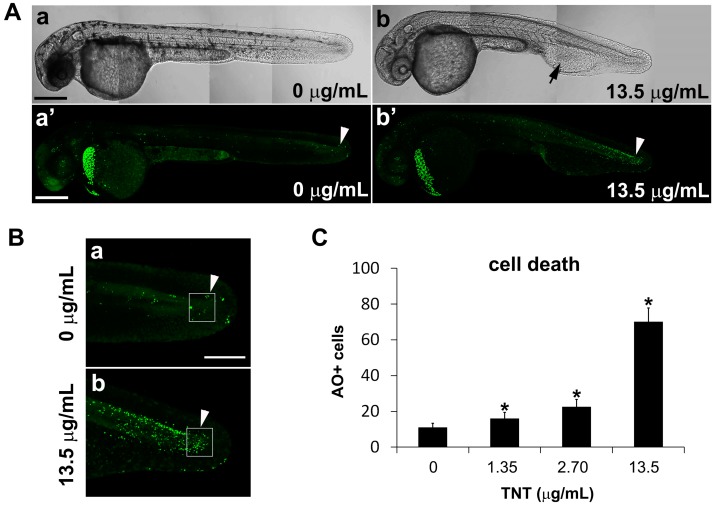
TNT in pink water increases the number of apoptotic cells in zebrafish embryos at 35 hpf. (**A**) Bright field images (**a**,**b**) and fluorescence images of acridine orange staining (**a′**,**b′**) by confocal microscopy show apoptosis in live whole embryos following treatment from 5–35 hpf with pink water containing TNT at 0 (**a**,**a′**) and 13.5 µg/mL (**b**,**b′**). Four tiled confocal images were combined to show the whole embryo in each case. Scale bar 500 µm. TNT treatment causes defective development of blood island and abnormal blood circulation (**b**, arrow) and more apoptosis in actively developing tissues of the tail (**b′**, arrowhead) compared with control (**a′**, arrowhead); (**B**) Magnified view of the tail regions of the embryos in (**A**) showing apoptotic cells (green dots); (**a**) is from **A** (**a′**) and (**b**) is from **A** (**b′**) respectfully. Scale bar 150 µm; (**C**) Number of acridine orange-positive apoptotic cells (AO + cells) in the boxed area in actively developing tail tissue (**B**, arrowhead) was counted at different concentrations of TNT using an Axioimager II fluorescence microscope. Cell death in TNT-treated embryos increased in a dose-dependent manner. *n* = 10 for each treatment, * *p* < 0.001.

To investigate the mechanism by which TNT causes developmental defects in zebrafish embryos, we stained live embryos with acridine orange dye to label apoptotic cells. Excitation of acridine orange with a 488 nm confocal laser allows the visualization of dying cells as green dots. Following exposure to pink water containing 13.5 µg/mL TNT from 5 hpf, apoptotic cells were visible as green dots in actively developing tissues throughout the body of 35 hpf embryos, including the caudal trunk and tail (Figure 5A,B). TNT treatment resulted in defective development of blood islands and abnormal blood circulation (Figure 5(Ab), arrow) and more apoptosis in actively developing tissues of the tail (Figure 5(Ab’), arrowhead) compared with control. The number of apoptotic cells in TNT-treated embryos was counted using an Axioimager II fluorescence microscope (*n* = 10 for each treatment). Statistical analysis shows TNT dose-dependently increased the number of apoptotic cells in the embryonic tail compared with controls (Figure 5C). The shortened body length of TNT-exposed embryos may be related to increased levels of cell death in the caudal trunk and tail.

## 3. Discussion

Human exposure to environmental contaminants from ammunition plants and demilitarization facilities is of global concern. Occupational or accidental exposure to explosive materials such as TNT (trinitrotoluene) by ingestion, inhalation, or dermal routes can also occur [1,2,3,4], and thus a full understanding of the toxicological effects of explosives on biological systems is desirable. This study adopted zebrafish embryogenesis as an in vivo assay for the detection of biological toxicity in samples of pink water. The zebrafish has numerous advantages for investigations of biotoxicity [28,29,30] and for modelling devastating human diseases [31,32]. Zebrafish embryos are small, can be obtained in large quantities, and develop externally, making them suitable for high-throughput assays. Crucially, zebrafish embryos are transparent, allowing live in vivo imaging, and genetically amenable, meaning that fluorescent transgenic lines can be generated to study specific developmental processes. Recent advances in state-of-the-art Single Plane Illumination Microscopy (SPIM)/light sheet microscopy open up new possibilities for sensitive in vivo toxicological bioassays: the results of this study demonstrate one such application.

Zebrafish embryos have been used previously for toxicity testing of various compounds and materials using conventional microscopy and molecular analyses: cardiac phenotypes such as abnormal looping, reduced chamber sizes and pericardial edema were shown when zebrafish embryos were treated with mycotoxin, polychlorinated biphenyls, or silica nanoparticles in early embryogenesis [33,34,35]. Here, we used zebrafish transgenic lines that robustly express fluorescent proteins in the developing heart, blood vessels and blood cells to assess TNT-mediated developmental toxicity using live 3D SPIM imaging at cellular resolution. Acquisition of 3D tissue data using confocal microscopy provides excellent resolution, but is limited by its speed and depth of imaging, meaning that in general, only small tissue samples can be scanned. Furthermore, photobleaching and phototoxicity limit long-term live imaging with confocal microscopes. SPIM overcomes these limitations, and thus is particularly well suited for live imaging studies of developmental processes. Spatial and temporal 3D mapping of fluorescently labeled tissues in zebrafish embryos can provide a framework on which to assess the toxic effect of drugs, chemicals or environmental pollutants [22,36].

Using both conventional and the state-of-the-art imaging technology, we report here the cellular changes in cardiac developmental defects resulting from TNT toxicity at different doses: pericardial edema, abnormal heart tube looping and especially hypoplasia of heart chambers, in addition to decrease in heart rate. SPIM permitted live visualization of endocardial endothelial cells expressing EGFP proteins in transgenic zebrafish embryos, and subsequent cell counting revealed hypoplasia in both ventricle and atrium even at very low concentration of TNT, 1.35 µg/mL. In addition, qPCR to quantitatively measure the molecular changes in the heart and blood development was sensitive enough to detect a significant decrease in the expression of the heart-specific genes *nkx2.5* [37] and *amhc* [38] in developing embryos exposed to TNT at the lowest concentration, 1.35 µg/mL. *nkx2.5* is an indicator of cardiac induction and an early marker of precardiac mesoderm, which is primarily expressed in ventricular progenitors at the eight-somite stage in zebrafish embryonic development [37]. *amhc* (atrial myosin heavy chain) is specifically expressed in atrial myocardium from the 19th somite stage [38]. Their expressions are specific to the heart in the whole embryo at the early stage and thus they have been used for qPCR makers to monitor the molecular change in early embryonic heart development [39]. Our qPCR data using these markers confirmed the significance of cardiac chamber hypoplasia following TNT exposure as shown in Figure 3B.

The hematotoxicity of TNT was previously reported in rats [5,6,7,8]. We observed developmental defects in blood islands and abnormal blood circulation after TNT exposure using state-of-the-art microscopy, and molecular analysis using real time PCR revealed decrease in the expression of *gata1,* an erythroid-specific transcription factor. *gata1* is a master regulator in erythrocyte development and its expression labels erythroid lineage in hematopoiesis during zebrafish embryonic development [40,41]. Our data suggest that the in vivo toxicity of environmental polutants such as TNT can be assessed using the zebrafish hematopoietic and vascular systems and can be confirmed quantitatively by both imaging and molecular analyses.

It has been reported that TNT toxicity caused changes in skin color and skin pigmentation in the adult bullfrog (*Lithobates catesbeiana*) [42]. Zebrafish pigmentation is initiated in the retinal epithelium and in the melanophores located dorsolaterally in the skin during embryogenesis [43]. Here, we observed a decrease in zebrafish pigmentation following treatment with TNT, even at low concentrations (Figure 1). Our finding that the severity of TNT′s toxicity was reduced when embryos were exposed to pink water at times later than 38 hpf, after early embryonic events including cleavage, gastrulation and neurulation are complete (Figure 1B), suggests that TNT may affect early melanocyte development from neural crest cells in addition to later melanin synthesis and pigment localization in melanocytes.

## 4. Materials and Methods

### 4.1. Animals

Wild type (AB strain) and the transgenic reporter strains Tg(cmlc2:EGFP) [27] and Tg(gata1:DsRed/fli1a:EGFP) [44,45] were used in this study. Adult zebrafish were maintained at 27.5 °C in a 14/10 light/dark cycle. Embryos were raised in E3 medium (5 mM NaCl, 0.33 mM MgSO_4_, 0.33 mM CaCl_2_, and 0.17 mM KCl) at 28.5 °C. Embryos or larvae were staged by hours post fertilization (hpf) or days post fertilization (dpf), according to Kimmel et al. [43].

The animal protocols used in this work were evaluated and approved by the Animal Use and Ethic Committee of the Kangwon National University (Protocol KW-151112-1). They are in accordance with Institutional Animal Care and Use Committee (IACUC) guidelines and the National law for Laboratory Animal Experimentation (Law No. 18.611).

### 4.2. Chemicals and High Performance Liquid Chromatography (HPLC) Analysis

Pink water, TNT (trinitrotoluene), RDX (cyclotrimethylenetrinitramine) and HMX (cyclotetramethylene-tetranitramine) were obtained from the Agency for Defense Development (Daejeon, Korea). The concentration of each explosive was quantified using a YL-9100 HPLC (Young Lin Instrument Co., Anyang, Korea) equipped with a TC-C18 column (Agilent, Santa Clara, CA, USA). An injection volume of 50 µL solvent, containing water and methanol at 1:1 (*v*/*v*) was used with a column temperature of 30 °C and detection wavelength of 254 nm (Figure S1).

### 4.3. Treatments

Pink water was diluted in E3 media. TNT powder was dissolved in ethanol and diluted in E3 media to final 1% ethanol for treatment. Final TNT concentration was measured by HPLC. Pink water and TNT were added to embryos at 50% epiboly stage (5 hpf) without dechorionation in E3 media. For each experiment, 20 embryos were treated in a 30 mm petri dish in a final volume of 3 mL E3 media, then incubated at 28.5 °C until required for analysis.

### 4.4. Live Imaging of Zebrafish Embryos

Zebrafish embryos were initially observed using an Olympus SZX16 stereoscope and bright field images were captured using AxioCam GRC camera (Carl Zeiss, Overkochen, Germany). Tg(cmlc2:EGFP) embryos were mounted laterally and time-lapse images of the beating heart were captured at 25 °C over 1 min intervals using an Axioimager II fluorescence microscope and an AxioCam GRC camera. Time-lapse imaging of the beating heart was recorded at 25 °C for 1 min. Heart rate was measured for 1 min at 27 °C without anesthesia using a Leica S6 E Microscope (LEICA, Wetzlar, Germany).

For confocal microscopy or light-sheet microscopy, zebrafish embryos were anesthetized in E3 media with 0.02% Tricaine and mounted as previously described [22,46]. During live imaging, fluorescence images of the transgenic embryos were acquired at 10× and 20× magnification using a LSM780 NLO confocal microscope (Carl Zeiss, Overkochen, Germany) with 10× dry objective and 20× water immersion objective lens at Korea Basic Science Institute Chuncheon Center (Chuncheon, Korea). EGFP was detected using a 488 nm Ar-laser (500–550 nm excitation) and DsRed using a 561 nm HeNe laser (570–630 nm excitation) [46].

Fluorescence microscopy images were collected using a light-sheet microscope with W Plan-Apochromat 20X/1.0 UV-VIS (Lightsheet Z.1, Carl Zeiss, Germany) at Korea Basic Science Institute Chuncheon Center. GFP and RFP fluorescence was excited with 488 nm and 561 nm lasers, and emission was detected by a 500–545 nm band-pass (BP) filter and a 575–615 nm BP filter as previously described [22]. The temperature of the light-sheet microscope chamber was maintained at 26 °C during imaging.

All acquired LSM raw data obtained from imaging were processed using ZEN 2011 software (Carl Zeiss, Germany). Optical sections were merged by maximum intensity projection for each z-stack and stacked group of sections were merged to form a partial or a whole body image of each embryo. Three-dimensional images were reconstructed using ARIVIS software 2.10.4 (Carl Zeiss, Germany; Korea Basic Science Institute Chuncheon Center); each voxel was recognized by the power of each fluorescence signal.

### 4.5. Acridine Orange Staining

Embryos were dechorionated and anesthetized in E3 media containing 0.02% Tricaine (Sigma-Aldrich, St. Louis, MO, USA). Embryonic apoptosis by chemical toxicity was measured using the vital dye, Acridine orange (Sigma-Aldrich) after exposure to chemicals. The embryos were stained with 3 µg/mL Acridine orange in E3 media at 25 °C for 30 min with shaking in the dark, washed 3 times with E3 media, mounted in 2% methylcellulose and observed alive using a Zeiss LSM780 NLO confocal microscope (488 nm Ar-laser excitation and 500–560 nm emission filter, Carl Zeiss, Overkochen, Germany).

### 4.6. Quantitative Real-Time PCR (qPCR)

Total RNA was extracted from 36 hpf embryos treated with TNT (1.35 µg/mL) at 5 hpf using TRIzol (Invitrogen). First-strand cDNA was synthesized from 2.7 μg of total RNA using Superscript II Reverse Transcriptase (Invitrogen) in accordance with the manufacture’s protocol. The qPCR analysis was performed on an CFX96 Real-Time PCR system (Bio-Rad) using IQ SYBR Green Supermix (Bio-Rad) as described in the manufacture’s protocol. qPCR primers for nkx2.5 and amhc genes were synthesized and used as previously described [39] and *gata1* primers as previously shown [41]. Each mRNA level was normalized to β-actin mRNA and then normalized to that of the control group. The relative mRNA expression of target genes was calculated based on Livak’s method [47]. Each experiment was repeated 3 times using independent batches of embryos and 10 embryos were included in each treated group.

### 4.7. Data and Statistical Analysis

Results are expressed as mean ± standard deviation. One-way analysis of variance (ANOVA) followed by Dunnett′s test was used to compare differences between treatment groups. Statistical significance for qPCR data was determined by independent-samples *t*-test. All statistical analyses were performed using SPSS 23.0 software (SPSS Inc., Chicago, IL, USA).

## 5. Conclusions

In conclusion, we have demonstrated the utility of advanced in vivo imaging of zebrafish embryos as a biological assay of environmental pollutant toxicity. Using 3D SPIM, we observed and quantified specific developmental defects, particularly cardiac abnormalities, in zebrafish embryos exposed to pink water containing TNT. Quantitative real-time PCR analysis of tissue-specific gene expression confirmed TNT effects at the molecular level. We also observed dose-dependent inhibition of melanocyte development and melanin synthesis and elevated levels of apoptosis in TNT-treated embryos. High-speed 3D imaging of organs using SPIM provides a means to quantify the toxicity of pollutants at the cellular level and increase our understanding of biotoxicological mechanisms.

## Supplementary Materials

Supplementary materials can be found at www.mdpi.com/1422-0067/17/11/1925/s1.
