# Supplementary material for: 3D Visualization of Developmental Toxicity of 2,4,6-Trinitrotoluene in Zebrafish Embryogenesis Using Light-Sheet Microscopy
Source: Int J Mol Sci. 2016 Nov 17;17(11):1925. doi: 10.3390/ijms17111925 (PMC5133921; doi:10.3390/ijms17111925)
Supplement: Supplementary file 1 [file ijms-17-01925-s001.zip › ijms-150877-Supplementary Materials/ijms-150877 Figure S1.pdf]

Int. J. Mol. Sci. **2016**, *17*, 1925; doi:10.3390/ijms17111925

Supplementary Material: 3D Visualization of Developmental Toxicity of 2,4,6-Trinitrotoluene in Zebrafish Embryogenesis Using Light-Sheet Microscopy

Juneyong Eum, Jina Kwak, Hee Joung Kim, Seoyoung Ki, Kooyeon Lee, Ahmed A. Raslan, Ok Kyu Park, Md Ashraf Uddin Chowdhury, Song Her, Yun Kee, Seung-Hae Kwon and Byung Joon Hwang

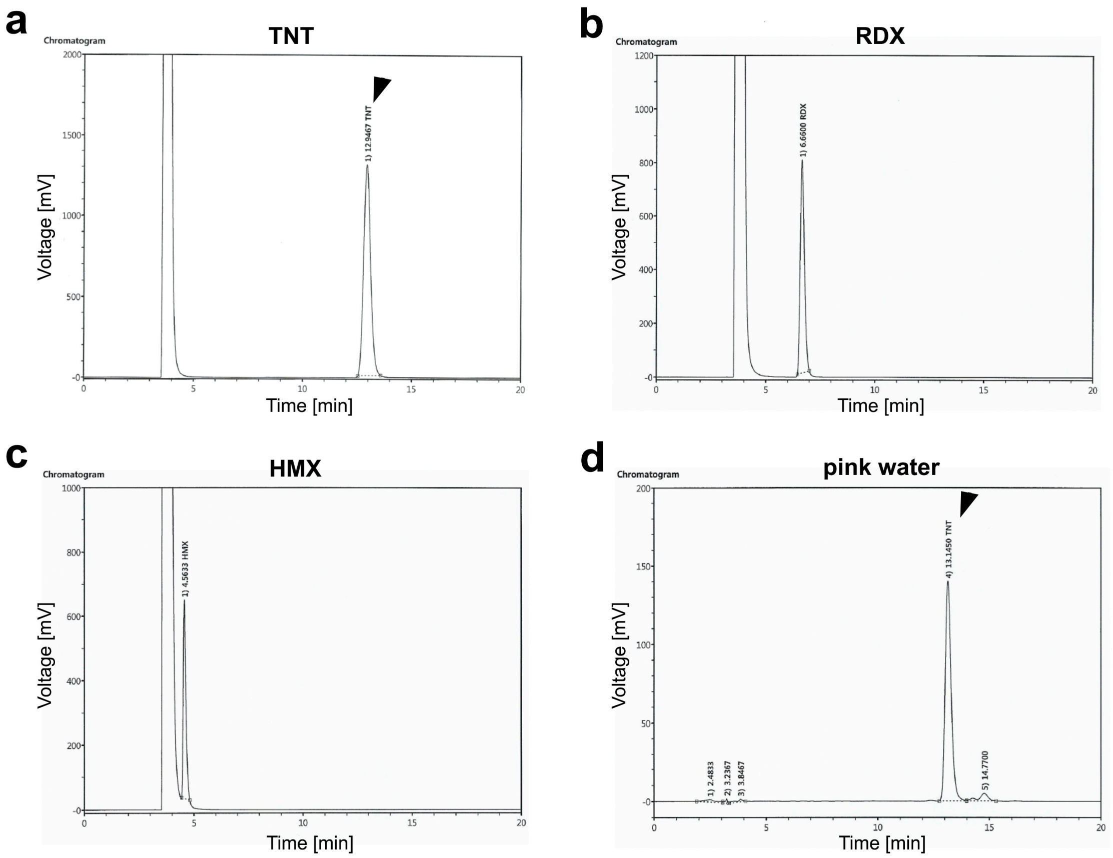

Figure S1. High performance liquid chromatography analysis of components in pink water. Retention times of trinitrotoluene (TNT) (**a**); cyclotrimethylenetrinitramine (RDX) (**b**); and cyclotetramethylene-tetranitramine (HMX) (**c**); which were dissolved in 1% acetone, were used as standards for the identification of pink water contaminants. The major peak in pink water was verified as TNT, as indicated by arrowheads (**d**).
